# Systemic Imidacloprid Affects Intraguild Parasitoids Differently

**DOI:** 10.1371/journal.pone.0144598

**Published:** 2015-12-14

**Authors:** Sally V. Taylor, Hannah J. Burrack, R. Michael Roe, Jack S. Bacheler, Clyde E. Sorenson

**Affiliations:** Department of Entomology, North Carolina State University, Raleigh, North Carolina, United States of America; Federal University of Viçosa, BRAZIL

## Abstract

*Toxoneuron nigriceps* (Viereck) (Hymenoptera, Braconidae) and *Campoletis sonorensis* (Cameron) (Hymenoptera, Ichneumonidae) are solitary endoparasitoids of the tobacco budworm, *Heliothis virescens* (Fabricius) (Lepidoptera, Noctuidae). They provide biological control of *H*. *virescens* populations in Southeastern US agricultural production systems. Field and greenhouse experiments conducted from 2011–2014 compared parasitism rates of parasitoids that developed inside *H*. *virescens* larvae fed on tobacco plants treated with and without imidacloprid. The parasitoids in our study did not have a similar response. *Toxoneuron nigriceps* had reduced parasitism rates, but parasitism rates of *C*. *sonorensis* were unaffected. Preliminary data indicate that adult female lifespans of *T*. *nigriceps* are also reduced. ELISA was used to measure concentrations of neonicotinoids, imidacloprid and imidacloprid metabolites in *H*. *virescens* larvae that fed on imidacloprid-treated plants and in the parasitoids that fed on these larvae. Concentrations were detectable in the whole bodies of parasitized *H*. *virescens* larvae, *T*. *nigriceps* larvae and *T*. *nigriceps* adults, but not in *C*. *sonorensis* larvae and adults. These findings suggest that there are effects of imidacloprid on multiple trophic levels, and that insecticide use may differentially affect natural enemies with similar feeding niches.

## Introduction

Systemic insecticide applications have a low risk of topical exposure to natural enemy populations because toxicants are transported inside of treated plant tissue [1]. However, there is a potential for food-chain effects when predators feed on contaminated prey [2]. Toxicants that transfer in this manner, including insecticides, affect the interactions between plants, herbivores and predators [3].

Insect parasitoids consume the body of their host and may be exposed to intoxicated prey. Parasitoids’ physiological and metabolic traits often make them more susceptible to toxicants than their hosts [4]. A tomato alkaloid, α-Tomatine, incorporated into the diet of *Helicoverpa zea* (Boddie) larvae decreases the survival and health of its parasitoid *Hyposoter exiguae* (Viereck) despite a tolerance to this toxin in the host [5]. Similarly, nicotine in the diet of *Manduca sexta* (Linnaeus) and *Spodoptera frugiperda* (J. E. Smith) transfers to parasitoid tissues and negatively affects survival and development of parasitoids *Cotesia congregata* (Say) and *Hyposoter annulipes* (Cresson) [6]. Parasitized hosts may also respond differently to toxicants compared to non-parasitized hosts; the LD50 of nicotine is lower for *Heliothis virescens* (Fabricius) larvae parasitized by *Campoletis sonorenis* (Cameron) [7]. In some cases, toxicants enhance the performance of parasitoids. *Bacillus thuringiensis* toxins administered to *H*. *virescens* in sublethal doses can aid its parasitoids by lengthening the amount of time the host spends in the stage vulnerable to parasitoid attack [8].

**Fig 1:**
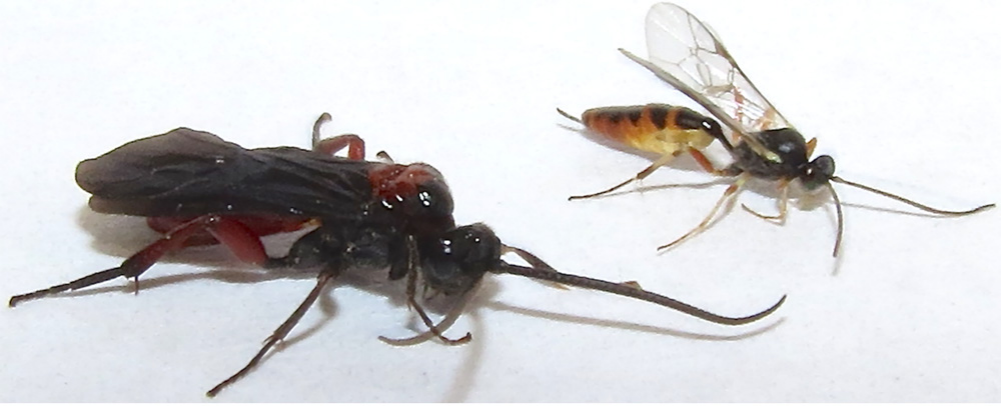
Adult female parasitoids. Adult females of *C*. *sonorensis* (right) and *T*. *nigriceps* (left).

Parasitoid species that coexist in the same environment and on the same resources compete to discover and exploit hosts [9]. *Campoletis sonorensis* and *Toxoneuron nigriceps* (Viereck) are solitary endoparasitoids of *H*. *virescens* larvae (Fig 1). They occur in overlapping regions of the southeastern United States and are found in cotton and tobacco cropping systems as well as non-crop hosts of *H*. *virescens*. Adult females of both species prefer to lay eggs in third instar larvae [10–14]. *Campoletis sonorensis* is a generalist capable of utilizing over 20 species of Noctuid hosts [14]. The host stops growing and feeding almost immediately following parasitism by *C*. *sonorensis* [15]. *Toxoneuron nigriceps* is a specialist and only completes development in *H*. *virescens* and *Heliothis subflexa* [16], [17], [10]. The host continues to eat and digest plant tissue, at a reduced rate, for up to 6 days following parasitism by *T*. *nigriceps* [18]. Parasitoids contribute significantly to biological control of *H*. *virescens* populations [19]. The percent of larvae parasitized by *T*. *nigriceps* and *C*. *sonorensis* has been reported between 50% and 100% in SE cropping systems [10], [16], [17], [20], [21].

Imidacloprid (Bayer CropScience, Monheim, Germany) is a widely-used agricultural insecticide that comes in a variety of formulations, is highly effective against piercing-sucking pests, and has a low mammalian toxicity [22–24]. Imidacloprid has the same target as nicotine within the insect nervous system [22], yet it varies in its toxicity to tobacco-feeding insects. Imidacloprid is used for management of thrips, aphids and flea beetles in flue-cured tobacco, but it has no insecticidal activity against tobacco-feeding lepidopteran pests, including *H*. *virescens*, because of their tolerance to similar compounds, like nicotine, in their diet [25], [26].

Imidacloprid can have lethal and sublethal effects on parasitoids exposed to environmental residues or to contaminated food sources [27–31]. However, imidacloprid is compatible with some host/parasitoid systems [32]. Since *H*. *virescens* larval mortality is not affected by imidacloprid treatments, the tobacco cropping system presents the opportunity to study how larval parasitoids respond to imidacloprid in the diet of their hosts. We hypothesized that imidacloprid moves from tobacco plants into *H*. *virescens* larvae through feeding and causes measurable effects on its parasitoids. In this study, we determined whether imidacloprid and its metabolites were present in the body of larval *H*. *virescens*, whether imidacloprid and its metabolites transferred from a host larva to its parasitoid, and determined any effects on the performance of parasitoids.

## Materials and Methods

### Field experiments

Field experiments were conducted from 2012–2013 at the Lower Coastal Plains Research Station in Lenoir County, North Carolina, and the Upper Coastal Plains Research Station in Edgecombe County, North Carolina, to measure the rate of *H*. *virescens* parasitism by natural populations of parasitoid species in imidacloprid treated and untreated tobacco plants. No permits or approvals were required for this work. Research station are jointly owned and managed by the North Carolina Department of Agriculture and North Carolina State University for the purpose of providing a platform for agricultural research. Insects collected from these experiments were used to compare imidacloprid concentrations and parasitoid lifespan. There are no guidelines for the ethical treatment of invertebrates. All insects were treated humanely and efforts were taken to minimize the suffering of animals used in this research.

Flue-cured tobacco seedlings (*Nicotiana tabacum* L, var. NC71), were grown in the greenhouse, following organically acceptable production methods. In 2012, transplants were set on April 18 at the Lenoir County site and on April 30 at the Edgecombe County site. Fields were bordered by flue-cured tobacco at the Lenoir County site, and woodlands on 2 sides and maize (*Zea mays* L.) on 2 sides at the Edgecombe County site. In 2013, transplants were set on April 24 at the Lenoir County site and on April 26 at the Edgecombe County site. Fields were bordered by flue-cured tobacco at the Lenoir County site, and by flue-cured tobacco on 2 sides and cotton (*Gossypium hirsutum* L.) on 2 sides at the Edgecombe County site. A minimum of 4 buffer rows separated test fields from surrounding flue-cured tobacco; there were no buffer rows between test fields and woodlands, maize or cotton. There were no neonicotinoid treatments used in test fields for a minimum of a year prior to use. Standard agronomic practices for tobacco production in North Carolina were used with the exception that insecticides, other than the imidacloprid treatments, were not applied [33]. Insecticides were applied following the labeled directions.

Untreated plants were compared to systemic, soil applications of imidacloprid (Admire Pro, Bayer CropScience, Research Triangle Park, NC). Imidacloprid was applied at the recommended field rate for tobacco (23.65 ml/1000 plants) using two methods: (1) a greenhouse tray drench followed by an immediate wash-off with clean water; and (2) a transplant water drench. The greenhouse tray drench was applied ≤ 2 days prior to transplanting. The transplant water drench was applied at the time of transplant. Treatment plots consisted of eight 15.24 m rows spaced 1.22 m apart with 20–25 plants per row. Plots were arranged in a randomized complete block design. There were 12 plots (4 replicates per treatment) at each site.

*Heliothis virescens* larvae were censused twice weekly from prior to the initiation of the adult *H*. *virescens* moth flight, as measured by the appearance of adult moths and eggs in the field, until tobacco plants flowered. Larvae were collected when they attained a size corresponding to ≥ late 3^rd^ stadium as measured by a gauge of head capsule size [8] and a ratio of head capsule size to body size. Larvae were reared on artificial diet in 25-ml cups, maintained at ambient conditions between 21–25°C, 50–70% relative humidity, 13–14 hours light and observed daily for parasitism [8]. Successful parasitism was defined by emergence of a live wasp larva. Parasitism was unsuccessful if either the host larvae pupated or died pre-parasitoid emergence. Deceased *H*. *virescens* larvae were not dissected for parasitoid eggs or larvae. Wasp larvae were not evaluated for survival post-emergence. Wasps emerging from field-collected larvae were positively identified to species. *Campoletis sonorensis* and *T*. *nigriceps* were the only parasitoids identified.

A subset of 3^rd^ instar *H*. *virescens* larvae collected at 60 (±7) days following planting date were randomly selected to be tested for imidacloprid and metabolite concentrations. Testing was destructive and these larvae were not used to assess parasitism rates. Larvae were not dissected prior to testing and it is unknown whether these larvae also contained developing parasitoids. A total of 63 larvae were tested. There were 30 larvae used in 2012, 15 from each location (5 per treatment). There were 33 larvae used in 2013: 15 from Edgecombe County (5 per treatment) and 18 from Lenoir County (6 per treatment).

Parasitoids that emerged from 3^rd^ instar *H*. *virescens* larvae collected at 60 (±7) days following planting date were randomly selected to either be tested for imidacloprid and metabolite concentrations or to be measured for lifespan. Testing for imidacloprid and metabolites was destructive and these parasitoids were not used to assess lifespan. Priority was given to imidacloprid and metabolite testing when limited numbers of insects were available.

A total of 126 *T*. *nigriceps* larvae and 114 *T*. *nigriceps* adults were used for imidacloprid and metabolite testing. Fifty-five larvae were used in 2012: 36 from Edgecombe County (6 per treatment) and 19 from Lenoir County (6 untreated, 7 greenhouse drench and 6 transplant water drench). Seventy-one larvae were used in 2013: 52 from Edgecombe County (8 untreated, 17 greenhouse drench and 27 transplant water drench) and 19 from Lenoir County (6 untreated, 6 greenhouse drench and 7 transplant water drench). Fifty adults were used in 2012: 24 from Edgecombe County (8 per treatment) and 26 from Lenoir County (10 untreated, 8 greenhouse drench and 8 transplant water drench). Sixty-four adults were used in 2013: 44 from Edgecombe County (18 untreated, 10 greenhouse drench and 16 transplant water drench) and 20 from Lenoir County (6 untreated, 8 greenhouse drench and 6 transplant water drench).

There were 46 *C*. *sonorensis* larvae and 44 *C*. *sonorensis* adults used for imidacloprid and metabolite testing. Eighteen larvae were used in 2012: 14 from Edgecombe County (4 untreated, 6 greenhouse drench and 4 transplant water drench) and 4 from Lenoir County (2 untreated and 2 transplant water drench). Twenty-eight larvae were used in 2013, all from Edgecombe County (8 untreated, 10 greenhouse drench and 10 transplant water drench). Twelve adults were used in 2012: 8 from Edgecombe County (2 untreated, 4 greenhouse drench and 2 transplant water drench) and 4 from Lenoir County (2 untreated and 2 greenhouse drench). Thirty-two adults were used in 2013: 30 from Edgecombe County (10 per treatment) and 2 from Lenoir County (untreated only).

Pesticides were extracted using methods established for parasitoids and host larvae [34]. *Heliothis virescens* larvae were starved for ≥ 1 day prior to testing to minimize the amount of plant tissue in the digestive tract. Parasitoid larvae were tested ≤ 1 day following emergence from host. Parasitoid adults were tested ≤ 1 day following emergence from the pupal cocoon and were not provided with food or water. Insects were euthanized by placement in a -20°C laboratory freezer for 30 minutes. Insects were rinsed 3 times in distilled water and dried with clean sterile Kim Wipe^®^ lab tissues. *Heliothis virescens* larvae were homogenized in a 1:1 ratio by weight with distilled water and centrifuged at 12,000 x g (Eppendorf^®^ minispin^®^ Hamburg, Germany) for 2 minutes at room temperature. An aliquot of 40 μl of the resulting supernatant was added to 100 μl of distilled water. The same procedure was used for *T*. *nigriceps* larvae. For *C*. *sonorensis* larvae and adults of both species, 2 insects from the same treatment were homogenized and tested together in 40 μl of distilled water.

The prepared samples were measured for imidacloprid concentrations by enzyme-linked immunosorbent assay (ELISA) using the QuantiPlate^™^ Kit for Imidacloprid (ENVIROLOGIX, Portland, Me) following manufacturer’s instructions. The manufacturer-provided imidacloprid standards were used because imidacloprid used in field experiments was formulated for agricultural systems. ELISA has cross-reactivity with other neonicotinoid insecticides (thiamethoxam, thiacloprid, clothianidin and acetamiprid) and imidacloprid metabolites (imidacloprid olefin, desnitro imidacloprid and imidacloprid urea). Imidacloprid concentrations detected by ELISA are likely to be from applied treatments; however, concentrations should be understood to potentially include imidacloprid, imidacloprid metabolites and cross-reacting neonicotinoids. The precision of ELISA measurements using these species was validated by fortifying insect samples as prepared above with 3 known concentrations of imidacloprid (0.2 ppb, 1 ppb and 6 ppb) and measuring the rate of recovery [35]. ELISA plates were read using a SpectraMax^®^ Plus 384 Microplate Reader (Molecular Devices, Sunnyvale, CA).

Adult female parasitoids that successfully emerged from pupal cocoons were used to assess parasitoid longevity. Parasitoids were kept in individual pint-sized Bugdorm^®^ cages (Bioquip, Rancho Dominguez, CA), fed honey (North Carolina State University Apiculture, Raleigh, NC), provided with water, and maintained at 25 ± 1°C, ≥ 70% relative humidity, 14 hours light: 10 hours dark. Wasps were observed daily for mortality, and wasps that did not move or respond to touch were considered deceased.

There were 34 *T*. *nigriceps* and 10 *C*. *sonorensis* observed for longevity. For *T*. *nigriceps*, there were 15 in 2012: 3 from Edgecombe County (1 per treatment) and 12 from Lenoir County (3 untreated, 5 greenhouse drench and 4 transplant water drench); and there were 19 in 2013: 16 from Edgecombe County (6 untreated, 5 greenhouse drench and 5 transplant water drench) and 3 from Lenoir County (1 per treatment). For *C*. *sonorensis*, all insects were from Edgecombe County: 3 in 2012 (1 per treatment) and 7 in 2013 (2 untreated, 2 greenhouse drench and 3 transplant water drench).

### Greenhouse experiments

Greenhouse experiments to measure the rate of *H*. *virescens* parasitism in treated and untreated tobacco plants were conducted from 2011–2013. Parasitoids used in greenhouse experiments were from laboratory colonies of *C*. *sonorensis* and *T*. *nigriceps* that were established from wasps collected in tobacco fields in Lenoir County and Edgecombe County, North Carolina in 2011. *Heliothis virescens* larvae were obtained from research colonies maintained at North Carolina State University, Raleigh, North Carolina. Adult parasitoids were kept in 29.8cm x 29.8cm bugdorm^®^ cages (Bioquip, Rancho Dominguez, CA) at approximately a 1:1 female to male ratio. Parasitoids were provided with honey (North Carolina State University Apiculture, Raleigh, NC) placed on petri dishes covered with a fine mesh screen to prevent wasps from becoming entrapped. Water was provided in a 50 mL glass Erlenmeyer flask with a dental wick and cotton ball inserted in the top to provide a drinking source and prevent drowning. The honey and water provided were not tested for imidacloprid because no wasps that had access to these materials were used to measure imidacloprid concentrations. Colonies were maintained at 25 ± 1°C, ≥ 70% relative humidity, 14 hours light; 10 hours dark.

Flue-cured tobacco seedlings (*Nicotiana tabacum* L, var. NC71) were grown under approved organic production methods in the greenhouse and transferred to 3-gallon plastic pots when 15–25cm tall. Soil used was Fafard^®^ 4P Mix. Plants treated with imidacloprid applied as a systemic soil-drench (Admire Pro, Bayer CropScience, Research Triangle Park, NC) at the label rate of 23.65ml/1000 plants were compared to untreated plants. Insecticide solutions were applied in 118 ml of water as a transplant water drench to the crown of the seedling at planting. Treatments were arranged in a randomized complete-block design. Mean greenhouse temperature was maintained at ≥ 25±3°C. Plants were watered 3–4 times a week as needed and not to overflowing.

Neonate *H*. *virescens* larvae were placed on plants 30–35 days following transplant to simulate the timing of the first field infestations. Larvae were placed on plants ≤ 1 day following their emergence using synthetic paint brushes (Simply Simmons Round Brush Size 1, Michaels Stores, Inc., Irving, TX). Plants were covered with custom-made organza bags to prevent movement of larvae because larvae were observed to move between plants on the same table. Late 2^nd^ instar larvae were collected after 7 (± 2) days and parasitized by wasps in the laboratory. Second instar larvae were chosen to ensure all larvae used were accepted by female parasitoids; some large 3^rd^ instar larvae repelled *C*. *sonorensis* females in preliminary experiments. Larvae were placed into 100 x 25 mm plastic petri dishes (Thomas Scientific, Swedesboro, NJ) with 2 female wasps and observed for wasp ovipositor insertion into larvae to ensure successful parasitism. Wasp pairs were randomly assigned to larvae in each treatment and were exposed to a maximum of 5 larvae per pair. Larvae were immediately returned to the plants of their origin and allowed to continue development. Larvae were observed daily starting at 7 days following parasitism for those parasitized by *C*. *sonorensis* and at 10 days following parasitism for those parasitized by *T*. *nigriceps*. Successful parasitism was defined by emergence of a live wasp larva. Wasp larvae were not evaluated for survival post-emergence. Parasitism was unsuccessful if either the host larva pupated or died pre-parasitoid emergence. Deceased *H*. *virescens* larvae were not dissected for parasitoid eggs or larvae. The experiment was replicated 4 times with different plants and different generations of insects. There were 15 plants used for each combination of parasitoid species and treatment for a total of 60 plants per replicate.

Greenhouse experiments to measure imidacloprid and metabolite concentrations in parasitized *H*. *virescens* larvae were conducted from 2013–2014. Tobacco seedlings were organically-grown, transplanted into pots, treated with insecticides and arranged into treatments as described above. The same greenhouse conditions, materials and watering protocols were maintained. Plants were treated with imidacloprid applied as a systemic soil-drench at two rates, the label rate (23.65ml/1000 plants) and 2 times the label rate (47.3ml/1000 plants), and were compared to untreated plants. Rates were selected to test the relationship between the amount of imidacloprid applied to the plant and the concentration of imidacloprid measured in the larvae. *Heliothis virescens* neonate larvae were placed on plants, confined with bags and parasitized as 2^nd^ instars by wasps in the laboratory as described above. Larvae were returned to their plant of origin and confined by bags for 6 days. Larvae were collected on day 6 following parasitism and tested for imidacloprid 7 days following parasitism using the ELISA protocol for *H*. *virescens* larvae as described above. Larvae were starved 24 hours prior to testing. The experiment was replicated 3 times with different plants and different generations of insects. There were 10 plants used for each combination of parasitoid species and treatment for a total of 60 plants per replicate. Larvae without signs of parasitism and deceased larvae were not tested. Larvae were determined to be parasitized by comparing their size and movement with non-parasitized larvae maintained in the same greenhouse conditions [36], [37]. There were 52 host larvae parasitized by *T*. *nigriceps* (12 untreated, 20 label rate and 20 2 x label rate) and 64 host larvae parasitized by *C*. *sonorensis* (24 untreated, 22 label rate and 18 2 x label rate) tested for imidacloprid and metabolite concentrations.

### Data analysis

Data were analyzed using SAS version 9.3 (SAS Institute, Cary, NC). Field data were subjected to analysis of variance (ANOVA) using the GLIMMIX procedure. Data from field experiments were pooled for analysis. Trends in parasitism rates and imidacloprid and metabolite concentrations within collection dates, locations and years were in agreement with results of the combined analysis; using a combined analysis did not change the significance of any variable. The incidence of parasitism, even in the same location, is known to fluctuate naturally between years [38]. Since this study spans two years and two sites, the variance attributable to the site was difficult to isolate from the variance attributable to the year. The variable “environment” was created to include both location and year. Data on parasitism rates for each species were analyzed separately because their rates of parasitism have differed historically [8], [17]. Explanatory variables used for analysis of field data included treatment, number of days between planting and collection date, replication and environment. The variables environment, replication and the number of days between planting and collection date were random. The variable of adult weight was added to the model for female wasp longevity. Data from greenhouse experiments were analyzed via the MIXED procedure. Explanatory variables included treatment and replication. Replication was treated as random. Data for both species of parasitoid were analyzed together for the experiment on imidacloprid and metabolite concentrations in parasitized *H*. *virescens* larvae because this experiment was designed to test how concentrations may be affected by parasitoid species. Means for all tests were separated using the Tukey-Kramer method.

## Results

### Field experiments

The parasitoids in our study did not have similar responses to larvae fed on imidacloprid-treated plants. There was a significant effect of imidacloprid treatment on the rate of parasitism by *T*. *nigriceps* (Table 1; df = 2, 268: F = 9.56: P = 0.0001). The mean percent parasitism in larvae from untreated plants (47.94 ± 3.6) was over 50% higher than the mean percentages from the greenhouse drench (30.27 ± 3.4) and the transplant water drench (28.38 ± 4.9). There were no differences in parasitism between imidacloprid applications. Environment was also significant in the model (df = 3, 268: F = 22.84: P<0.0001). There was no significant effect of replicate (df = 3, 268: F = 0.25: P = 0.8621) or number of days between planting and collection date (df = 16, 268: F = 0.96; P = 0.5012).

**Table 1:** Mean percent of field-collected *H*. *virescens* larvae parasitized by *C*. *sonorensis* and *T*. *nigriceps* from flue-cured tobacco (*Nicotiana tabacum*) plots in Edgecombe and Lenoir Counties, North Carolina (2012–2013) under different treatments of imidacloprid.

Treatment	% parasitism (±SEM)	
	*C*. *sonorensis*	*T*. *nigriceps*
Untreated	11.43 (3.4)	47.94 (3.6)*
Imidacloprid (23.65ml/1000 plants) applied as a greenhouse drench	5.84 (1.5)	30.27 (3.4)
Imidacloprid (23.65ml/1000 plants) applied as a transplant water drench	7.24 (1.8)	28.38 (4.9)

*denotes means significantly different within a column at α = .05

Imidacloprid treatment had no significant effect on the rate of parasitism by *C*. *sonorensis* (Table 1; df = 2, 268: F = 0.08: P = 0.9301). There was also no significant effect of environment (df = 3, 268: F = 1.4: P = 0.2432), replicate (df = 3, 268: F = 0.45: P = 0.7175) or number of days between planting and collection date (df = 16, 268: F = 1.21; P = 0.3065).

Concentrations of neonicotinoids, imidacloprid and/or imidacloprid metabolites were detected in *H*. *virescens* larvae, *T*. *nigriceps* larvae and *T*. *nigriceps* adults (Table 2). Concentrations differed significantly by imidacloprid treatment [*H*. *virescens* larvae (df = 2, 54: F = 487.26: P<0.0001); *T*. *nigriceps* larvae (df = 2, 117: F = 15.81: P<0.0001); *T*. *nigriceps* adults (df = 2, 105: F = 8.08: P = 0.0005)]. There were lower concentrations in insects from untreated plants [*H*. *virescens* larvae (0.69 ± 0.07 ppb); *T*. *nigriceps* larvae (0.09 ± 0.02 ppb); *T*. *nigriceps* adults (0.22 ± 0.01 ppb)] compared to insects from plants treated with imidacloprid applied in the greenhouse [*H*. *virescens* larvae (7.66 ± 0.02 ppb); *T*. *nigriceps* larvae (0.55 ± 0.07); *T*. *nigriceps* adults (0.31 ± 0.03)] and applied in transplant water [*H*. *virescens* larvae (7.91 ± 0.01 ppb); *T*. *nigriceps* larvae (0.70 ± 0.06); *T*. *nigriceps* adults (0.37 ± 0.03)]. Concentrations were not affected by environment [*H*. *virescens* larvae (df = 3,54: F = 0.99: P = 0.4027); *T*. *nigriceps* larvae (df = 3, 117: F = 1.28: P = 0.2848); *T*. *nigriceps* adults (df = 3, 105: F = 0.40: P = 0.7525)] or replicate [*H*. *virescens* larvae (df = 3, 54: F = 0.05: P = 0.9847); *T*. *nigriceps* larvae (df = 3, 117: F = 0.36: P = 0.7808); *T*. *nigriceps* adults (df = 3, 105: F = 0.69: P = 0.5612)]. There were no measurable concentrations in *C*. *sonorensis* larvae or adults.

**Table 2:** Mean concentration of neonicotinoids and metabolites (in ppb) in field-collected *H*. *virescens* larvae, *T*. *nigriceps* larvae and *T*. *nigriceps* adults from flue-cured tobacco (*Nicotiana tabacum*) plots in Edgecombe and Lenoir Counties, North Carolina (2012–2013) under different treatments of imidacloprid.

Treatment	Concentration (±SEM) by species (life stage)		
	*H*. *virescens* (larvae)	*T*. *nigriceps* (larvae)	*T*. *nigriceps* (adults)
Untreated	0.69 (0.07)*	0.09^†^ (0.02)*	0.22^†^ (0.01)*
Imidacloprid (23.65ml/1000 plants) applied as a greenhouse drench	7.66 (0.02)	0.55 (0.07)	0.31 (0.03)
Imidacloprid (23.65ml/1000 plants) applied as a transplant water drench	7.91 (0.01)	0.70 (0.06)	0.37 (0.03)

*denotes means significantly different within a column at α = .05

^†^denotes concentration below the limit of quantification (0.3)

The lifespan of *T*. *nigriceps* adult females differed significantly between treatments with females from larvae on untreated plants having a 10–12 day longer lifespan compared to females that emerged from larvae on plants treated with imidacloprid (Table 3; df = 2,16: F = 5.77: p = 0.0130). There were no differences in lifespan between the two imidacloprid applications. Insect weight also significantly influenced survival, with larger insects having longer lifespans (df = 5,16: F = 5.56: p = 0.0037). Lifespans were not affected by environment (df = 3,16: F = 1.30: p = 0.3072) or replicate (df = 3,16: F = 1.46: p = 0.2620). The interaction of treatment and weight was not significant (df = 4, 16: F = 0.71: p = 0.5971). However, the number of wasps in this study was limited and results from these data are considered preliminary. There were not enough *C*. *sonorensis* available for statistical analysis.

**Table 3:** Mean lifespan (in days) of adult female *T*. *nigriceps* that emerged from *H*. *virescens* larvae collected from flue-cured tobacco (*Nicotiana tabacum*) plots in Edgecombe and Lenoir Counties, North Carolina (2012–2013) under different treatments of imidacloprid.

Treatment	Lifespan (±SEM)
Untreated	39.8 (3.9)*
imidacloprid (23.65ml/1000 plants) applied as a greenhouse drench	27.9 (2.8)
Imidacloprid (23.65ml/1000 plants) applied as a transplant water drench	28.3 (2.8)

*denotes means significantly different at α = .05

### Greenhouse experiments

There were significant differences in the rate of parasitism by *T*. *nigriceps* in *H*. *virescens* larvae caged on untreated plants compared to larvae on imidacloprid treated plants (Table 4; df = 1, 3: F = 15.53: P = 0.0291). The mean percent parasitism in larvae from untreated plants (62.27 ± 5.2) was over 50% higher than the mean percent from the transplant water drench (37.00 ± 6.6). Parasitism rates were not affected by replicate (df = 3, 3: F = 2.62: P-0.2246). There were no differences in the rate of parasitism by *C*. *sonorensis* by treatment (df = 1, 7: F = 1.08: P = 0.3395) or replicate (df = 3, 3: F = 1.91: P = 0.3042).

**Table 4:** Mean percent of *H*. *virescens* larvae from flue-cured tobacco (*Nicotiana tabacum*) treated with imidacloprid applied as a transplant water drench in the greenhouse (2011–2013) that were successfully parasitized by *C*. *sonorensis* and *T*. *nigriceps*.

Treatment	% parasitism (±SEM)	
	*C*. *sonorensis*	*T*. *nigriceps*
Untreated	48.75 (10.6)	62.27 (5.2)*
Imidacloprid (23.65ml/1000 plants) applied as a transplant water drench	32.25 (7.0)	37.00 (6.6)

*denotes means significantly different within a column at α = .05

Concentrations of neonicotinoids, imidacloprid and/or imidacloprid metabolites were detected in parasitized *H*. *virescens* larvae (Table 5). Concentration levels differed by imidacloprid rate (df = 2,108: F = 288.31: P<0.0001) and parasitoid species (df = 2,108: F = 162.04: P<0.0001). The interaction of the imidacloprid rate and the parasitoid species was also significant (df = 2,108: F = 35.32: P<0.0001). Concentrations were not affected by replicate (df = 2, 108: F = 2.20: P = 0.1157). Concentrations detected in parasitized larvae were higher when a higher rate of imidacloprid was applied to the plant, and at each application rate, concentrations were higher in larvae parasitized by *T*. *nigriceps* than in larvae parasitized by *C*. *sonorensis*.

**Table 5:** Mean concentration of neonicotinoids and metabolites (in ppb) in *C*. *sonorensis* and *T*. *nigriceps* parasitized *H*. *virescens* larvae from flue-cured tobacco (*Nicotiana tabacum*) treated with different rates of imidacloprid applied as a transplant water drench in the greenhouse.

Treatment	Concentration (±SEM)	
	*H*. *virescens* larvae parasitized by *C*. *sonorensis*	*H*. *virescens* larvae parasitized by *T*. *nigriceps*
Untreated	0.246 (0.02)*	0.338 (0.07)*
imidacloprid (23.65ml/1000 plants) applied as a transplant water drench	2.251 (0.38)* ^†^	8.268 (0.54)* ^†^
imidacloprid (47.3ml/1000 plants) applied as a transplant water drench	6.958 (0.58)* ^†^	12.961 (0.25)* ^†^

*denotes means significantly different within a column at α = .05

^†^denotes means significantly different within a row at α = .05

## Discussion

### Insecticide testing

We measured concentrations of neonicotinoids, imidacloprid and imidacloprid metabolites in the bodies of *H*. *virescens* larvae that fed on treated tobacco plants and in parasitoids that developed from these larvae. Our results demonstrate that imidacloprid can move between multiple trophic levels of the agroecosystem. Tritrophic movement of imidacloprid has also been demonstrated between soybean plants, herbaceous slugs and predators [39].

Concentrations of neonicotinoids, imidacloprid and imidacloprid metabolites were detected in insects from untreated plants in our field experiments, although these concentrations were significantly lower than those detected in insects from treated plants. It is possible that this finding may be the result of carry-over of neonicotinoids, imidacloprid and imidacloprid metabolites from agricultural usage in previous years. These materials were detected in the water supply at both field locations (unpublished data) indicating the persistence of these materials in the research station environment. It is also possible for a matrix effect to occur with the ELISA test when insect hemolymph is insufficiently diluted and this effect will falsely indicate higher imidacloprid concentrations [34]. However, when imidacloprid rates applied to the plants in the greenhouse were doubled, there was a corresponding increase in the concentrations detected in insects from these plants, validating the use of this method for comparative, if not quantitative, purposes. Concentrations were higher in *H*. *virescens* larvae parasitized by *T*. *nigriceps* from greenhouse experiments (Table 5) than in *H*. *virescens* larvae collected from the field (Table 2). We hypothesize that insecticide concentrations in greenhouse plants, where humidity, temperature and precipitation were controlled, were higher than in plants exposed to natural conditions. Differences in UV light, root growth, humidity and soil nitrogen content all contribute to differences in leaf chemistry between pot-bound and field-grown plants [40].

### Effects on parasitism

Our results demonstrate that parasitoid species that inhabit the same agricultural environments and utilize the same host differ in their response to hosts fed on imidacloprid-treated tobacco plants. Parasitism rates of *T*. *nigriceps* are reduced by over 50% when host larvae are fed on treated plants. However, parasitism rates of *C*. *sonorensis* are unchanged. Imidacloprid can reduce the ability of female parasitoids to find hosts by altering plant volatiles [41], or by causing irritation or locomotive depression [29]. These effects could be partially responsible for decreasing *H*. *virescens* parasitism by *T*. *nigriceps*. However, parasitism was also reduced in greenhouse experiments where insertion of the parasitoid ovipositor into the host larva occurred, indicating that effects on *T*. *nigriceps* parasitism is the result of a change in host acceptance by the female parasitoid following ovipositor insertion or host suitability for the developing parasitoid.

A change in host suitability is strongly supported by the presence of trace amounts of imidacloprid and/or metabolites in the bodies of larval and adult parasitoids. Yet, it is possible that imidacloprid-fed hosts are rejected by female wasps after probing. We did not dissect *H*. *virescens* larvae for parasitoid eggs or larvae in these experiments. In prior studies, dissections of field-collected larvae have poorly estimated the instance of *H*. *virescens* parasitism [10]. Dissections of only those deceased host larvae revealed few parasitoid eggs or larvae regardless of imidacloprid treatment (unpublished data). It is possible that parasitoids were either missed during dissection or too degraded to identify. Parasitized hosts that do not pupate will live for a period of up to 1 month until they starve and desiccate; encapsulated parasitoids may or may not be present in these hosts on dissection [37]. Immunosuppression from teratocytes and polydnaviruses will prevent *H*. *virescens* from successfully developing into an adult in the absence of an immature parasitoid, but pupation may or may not still occur [42]. We did not rear *H*. *virescens* pupae to assess survival rates. Detailed examinations are needed at each developmental time point to fully understand how imidacloprid affects *T*. *nigriceps* parasitism.

Different responses to insecticide exposure are possible between adult and immature parasitoids and between 2 parasitoid species; this phenomenon has occurred with other insecticidal classes [43]. Previously, we showed that *C*. *sonorensis* adults are more susceptible than *T*. *nigriceps* adults to topical imidacloprid exposure [44]; and, based on this toxicity, we expected *T*. *nigriceps* parasitism to be less affected by imidacloprid incorporated into the host diet. *Toxoneuron nigriceps* may yet prove to be the more robust species, but it is exposed to higher concentrations during its development. Insecticide and metabolite concentrations in *H*. *virescens* larvae parasitized by *T*. *nigriceps* were 4 times higher than concentrations in larvae parasitized by *C*. *sonorensis*. Differences in how *T*. *nigriceps* and *C*. *sonorensis* manipulate host feeding may be responsible for differences in concentration. *Heliothis virescens* larvae stop feeding almost immediately following parasitism by *C*. *sonorensis* [15], but larvae parasitized by *T*. *nigriceps* continue to eat and digest treated plants for up to 6 days [18]. Food travels slower through the digestive tracts of larvae parasitized by *T*. *nigriceps* [18], which may increase the absorption of ingested imidacloprid in the digestive tract. More work is needed on how imidacloprid effects the development of *T*. *nigriceps* larvae.

### Conclusions

The compatibility of insecticides and beneficial insects is an important consideration in integrated pest management. Insecticides that destroy natural enemies can be causal agents in pest resurgences and secondary pest outbreaks [45]. Here we report an instance of an insecticide that decreases the percentage of parasitoids that survive to parasitize following generations of a pest. However we cannot say that this decrease has effects on biological control of *H*. *virescens* or on the composition of beneficial insect populations.

The movement and fate of pesticides released into the environment is of concern to its inhabitants. *A priori* knowledge tells us that individual organisms that encounter toxic materials suffer metabolic stress from this exposure [46]. Here we provide empirical evidence that imidacloprid is capable of moving through trophic levels of the agroecosystem, and that this movement causes deleterious effects on some, but not all, beneficial species. The long-term consequences on the population dynamics of this predator-prey system, if any, are unknown.
